# Supplementary material for: Bronchoalveolar Lavage and Blood Markers of Infection in Critically Ill Patients—A Single Center Registry Study
Source: J Clin Med. 2021 Jan 29;10(3):486. doi: 10.3390/jcm10030486 (PMC7866381; doi:10.3390/jcm10030486)
Supplement: Supplementary file 1 [file jcm-10-00486-s001.pdf]

Supplemental tables

Supplemental Table S1: relevant species of bacteria and fungi found in BAL

Bacterial and fungal pathogens	count
Aspergillus species	18
Burkholderia cepacia	3
Burkholderia species	1
Citrobacter koseri	1
Enterobacter cloacae	8
Enterobacterales	1
Enterococcus faecalis	1
Enterococcus faecium	4
Escherichia coli	18
Haemophilus influenzae	1
Hyphomycetes	1
Klebsiella aerogenes	1
Klebsiella oxytoca	7
Klebsiella pneumoniae	9
Legionella pneumophila	5
Moraxella catarrhalis	1
Mycobacterium intracellulare	1
Mycobacterium tuberculosis	2
Pasteurella multocida	1
Pseudomonas aeruginosa	36
Raoultella (Klebsiella) ornithinolytica	2
Serratia marcescens	5
Staphylococcus aureus	20
Stenotrophomonas maltophilia	15
Streptococcus anginosus group	1
Streptococcus pneumoniae	3
Streptococcus pyogenes	1
Total number	167

Overview of the detected relevant bacterial and fungal microorganisms. Detection of multiple species in a single BAL was possible. All cases of detected relevant bacteria and fungi are included individually.

Supplemental Table S2: all bacteria and fungi found in BAL

Bacterial and fungal pathogens	count
Acrophialophora levis	1
Aspergillus fumigatus	17
Aspergillus nidulans	1
Burkholderia cepacia	3
Burkholderia species	1
Candida albicans	54
Candida dubliniensis	3
Candida glabrata	13
Candida guilliermondii	1
Candida krusei	3
Candida lusitaniae	6
Candida palmioleophila	1
Candida parapsilosis	6
Candida species	68
Candida tropicalis	9
Citrobacter koseri	1
Clavispora lusitaniae	1
Enterobacter cloacae	8
Enterobacterales	1
Enterococcus faecalis	1
Enterococcus faecium	4
Escherichia coli	18
Fusarium species	1
Haemophilus influenzae	1
Hyphomycetes	1
Klebsiella aerogenes	1
Klebsiella oxytoca	7
Klebsiella pneumoniae	9
Kluyvera ascorbata	1
Kluyveromyces marxianus	1
Lactobacillus fermentum	1
Lactobacillus rhamnosus	1
Legionella pneumophila	5
Moraxella catarrhalis	1
Mycobacterium intracellulare	1
Mycobacterium tuberculosis	2
Paecilomyces species	1
Pasteurella multocida	1
„Physiological flora“ [1]	77
Pseudomonas aeruginosa	36
Raoultella (Klebsiella) ornithinolytica	2
Saccharomyces cerevisiae	1
Serratia marcescens	5
„Replacement flora“ [2]	64

Staphylococcus aureus	20
Staphylococcus epidermidis	1
Stenotrophomonas maltophilia	15
Streptococcus agalactiae	1
Streptococcus pneumoniae	3
Streptococcus pyogenes	1
Streptococcus anginosus group	1
Wickerhamomyces anomalus	1
Yeasts	27
Total number	511

Overview of all detected bacterial and fungal microorganisms. Detection of multiple species in a single BAL was possible. All cases of detected bacteria and fungi are included individually.

[1] "Physiological flora" is a term reported back by the microbiological examiner. It was reported when microorganisms of the natural flora (likely contaminations) were found in lavage fluid. No further information about the found species was provided.

[2] "Replacement flora" was also reported by the microbiological examiner when microorganisms that typically colonize patients under antibiotic therapy had been detected and were suspected to be contaminants of the lavage fluid. In this case there was no further information about the exact species as well.

Supplemental figure S1: Markers of inflammation excluding BAL with clinically relevant fungi

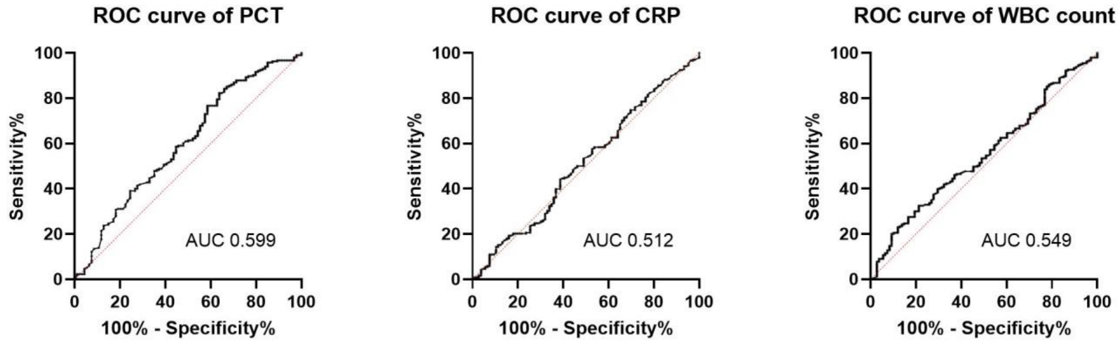

Markers of inflammation in the whole collective excluding the 18 patients with clinically relevant fungi in the BAL fluid. ROC curve showing the diagnostic values of PCT and CRP culture positive BAL with relevant bacteria. Abbreviations: BAL = bronchoalveolar lavage, WBC = while blood cell, CRP = C-reactive protein, PCT = procalcitonin.

Supplemental figure S2: Markers of inflammation using a more rigid definition of relevant bacteria

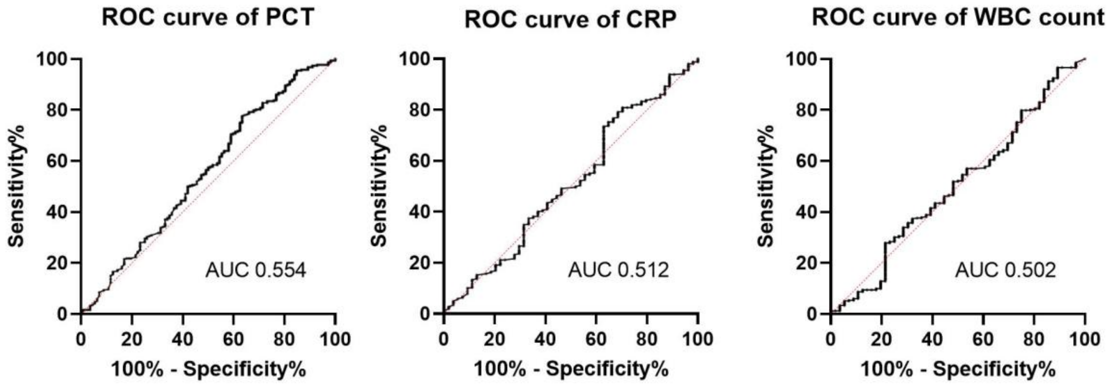

Markers of inflammation in the whole collective using a more rigid definition of relevant bacteria. ROC curve showing the diagnostic values of PCT and CRP culture positive BAL with relevant bacteria. Abbreviations: BAL = bronchoalveolar lavage, WBC = while blood cell, CRP = C-reactive protein, PCT = procalcitonin.
